# Solitary Fibrous Tumor of Pancreas With Unusual Features: A Case Report

**DOI:** 10.7759/cureus.10833

**Published:** 2020-10-06

**Authors:** Anoshia Afzal, Manuel Maldonado-Vital, Shahbaz Khan, Umar Farooque, Wenyi Luo

**Affiliations:** 1 Pathology, University of Oklahoma Health Sciences Center, Oklahoma City, USA; 2 Neurology, Dow University of Health Sciences, Karachi, PAK

**Keywords:** pancreas, pleura, patternless pattern, pleomorphism, solitary fibrous tumor, extrapleural, humans

## Abstract

Solitary fibrous tumor (SFT) is an uncommon fibroblastic neoplasm that is most commonly associated with the pleura but has also been reported in almost all anatomic sites. Although the majority of SFTs are benign, few cases follow a malignant clinical course and may recur and/or metastasize after several years of their original occurrence. Only 16 cases of pancreatic SFTs are reported so far, and only one has metastasized to lung and subcutis. Pancreatic SFT resembles more common neuroendocrine tumor and gastrointestinal stromal tumor (GIST) radiographically and is at times almost indistinguishable from GIST histologically. Diagnosis of SFTs particularly, if attempted on biopsied specimens, can be very challenging due to its rare occurrence and nondescript morphology. It is imperative to understand the pathological spectrum of this entity to avoid misdiagnosis. We report a case of pancreatic SFT in a 43-year-old male with some unusual morphologic and immunohistochemical features including pseudoangiomatous growth pattern, a hypercellular area demonstrating nuclear pleomorphism, and only focal positivity for cluster of differentiation (CD)34. These atypical features can pose even more diagnostic challenge by causing additional confusion with other malignancies like dedifferentiated liposarcoma and vascular tumors. The potential diagnostic pitfalls are discussed.

## Introduction

Solitary fibrous tumor (SFT) is a mesenchymal tumor that was initially identified in the pleura. Thereafter, extrapleural cases from various anatomic sites were reported. The etiology appears to be related to a paracentric inversion on chromosome 12q13, which creates an NGFI-A-binding protein 2-signal transducer and activator of transcription 6 (NAB2-STAT6) fusion gene [1].

Histologically, extrapleural SFTs appear similar to pleural SFT; but the prognosis and biological behavior are different as extrapleural SFTs are potentially more aggressive, and management of each case needs to be individualized. Among the reported cases, most of the pancreatic SFTs presented as well-circumscribed, often partially encapsulated masses with typical features of an extrapleural SFT including the patternless architecture of fibroblast-like spindle cells with intervening thick bands of collagen, perivascular sclerosis, and prominent hyalinized (staghorn) vessels [2-7]. The case we present here demonstrates some atypical features that can be easily confused with other entities. Recognizing these atypical features is imperative for an accurate diagnosis.

## Case presentation

A 43-year-old male, with a known history of hypertension, seizures, and traumatic brain injury secondary to a motor vehicle accident 23 years ago, presented to our emergency department with a right-sided incarcerated inguinal hernia for which his workup was done, and computed tomography (CT) scan of the abdomen revealed a large, well-circumscribed solid, and centrally cystic mass (12.1 cm x 12.7 cm) in the head and neck region of the pancreas. The patient denied any symptoms related to the mass, and laboratory data were unremarkable. Gastrointestinal stromal tumor or neuroendocrine tumor was clinically suspected. Fine needle aspiration of the mass demonstrated a spindle cell neoplasm. However, no definitive diagnosis could be given. Whipple procedure was subsequently performed. The mass was grossly an encapsulated tumor with a variegated, tan-white, slightly whorled mass with areas of hemorrhage. The tumor was widely away from all the margins including the proximal/gastric margin, distal/duodenal margin, pancreatic neck margin, and ampulla of Vater.

**Figure 1:**
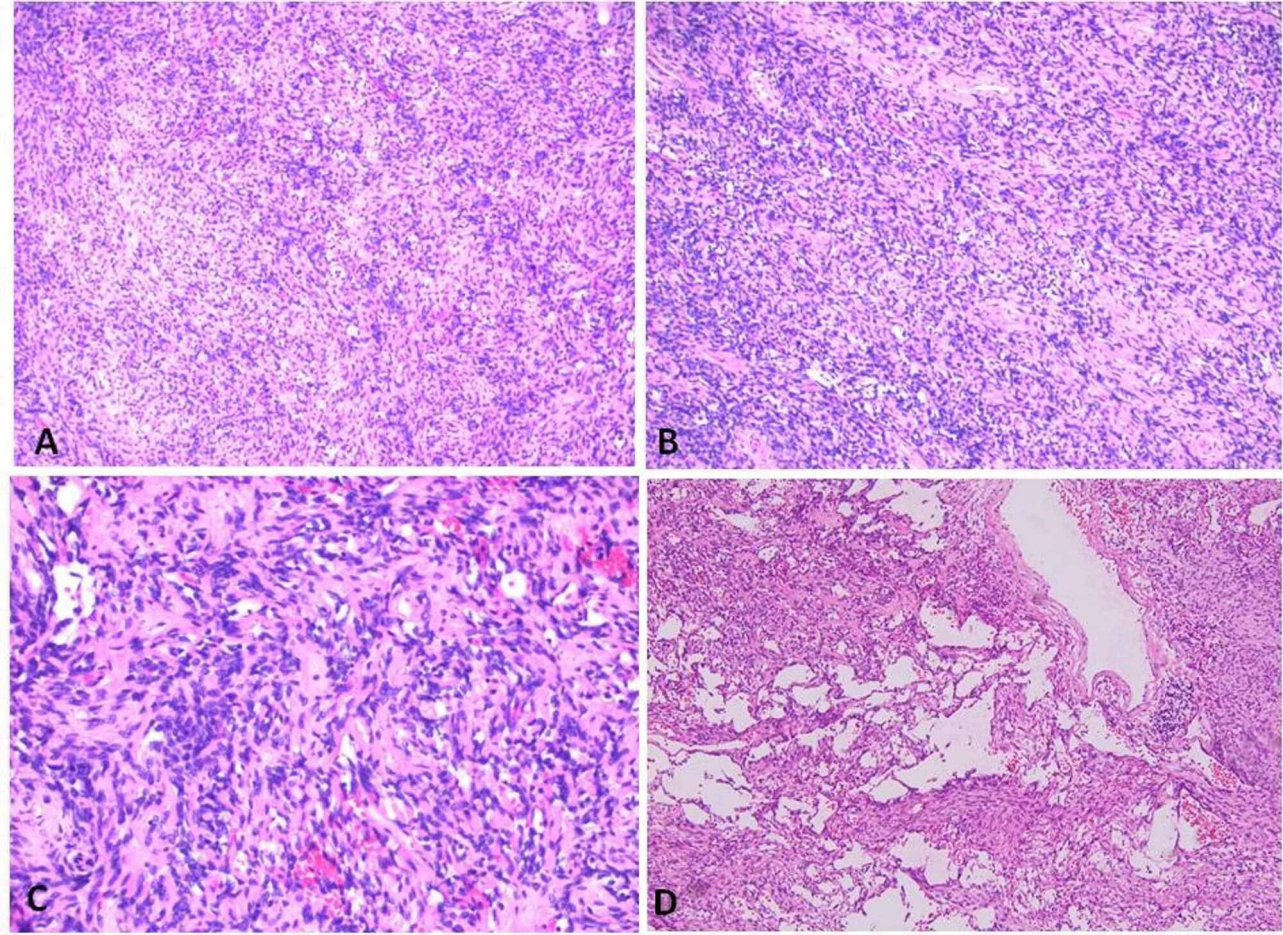
Hematoxylin and eosin staining shows a patternless pattern typical of SFT (A and B: 10x; C: 20x) and an atypical pseudoangiomatous area mimicking a vascular tumor (D: 10x). SFT: Solitary fibrous tumor.

Microscopic examination revealed a tumor that was composed of invasive sheets of spindle cells arranged in a patternless pattern (Figures 1A-1C) with intervening “ropey collagen”. Focal areas mimicking a vascular tumor (Figure 1D) were present.

**Figure 2:**
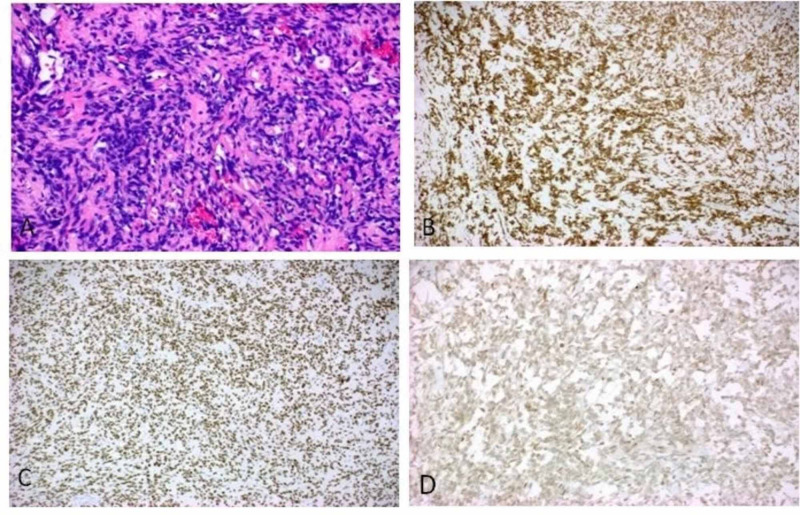
Hematoxylin and eosin staining shows a hypercellular area (A). The tumor cells are diffusely and strongly positive for Bcl 2 (B) and STAT6 (C). CD117 is unusually positive (D). Bcl: B-cell lymphoma. STAT: Signal transducer and activator of transcription. CD: Cluster of differentiation.

No characteristic staghorn vessels were identified. The cells had minimal cytoplasm, small elongated nuclei, and indistinct nucleoli. No adipocytic or heterologous differentiation was appreciated. Hypercellular zone (Figure 2A) was present at the periphery of the tumor where tumor cells demonstrated moderate pleomorphism; however, no increase in mitoses or necrosis was identified. No lymphovascular invasion was identified either. Hence, no morphologic evidence of malignant transformation or dedifferentiation was present. Immunohistochemical stains were performed to characterize the tumor. The tumor was diffusely and strongly positive for B-cell lymphoma (Bcl) 2 and STAT6 (Figures 2B, 2C) and focally positive for cluster of differentiation (CD)99, confirming the diagnosis of an SFT. The tumor was unusually positive for CD117 (Figure 2D).

**Figure 3:**
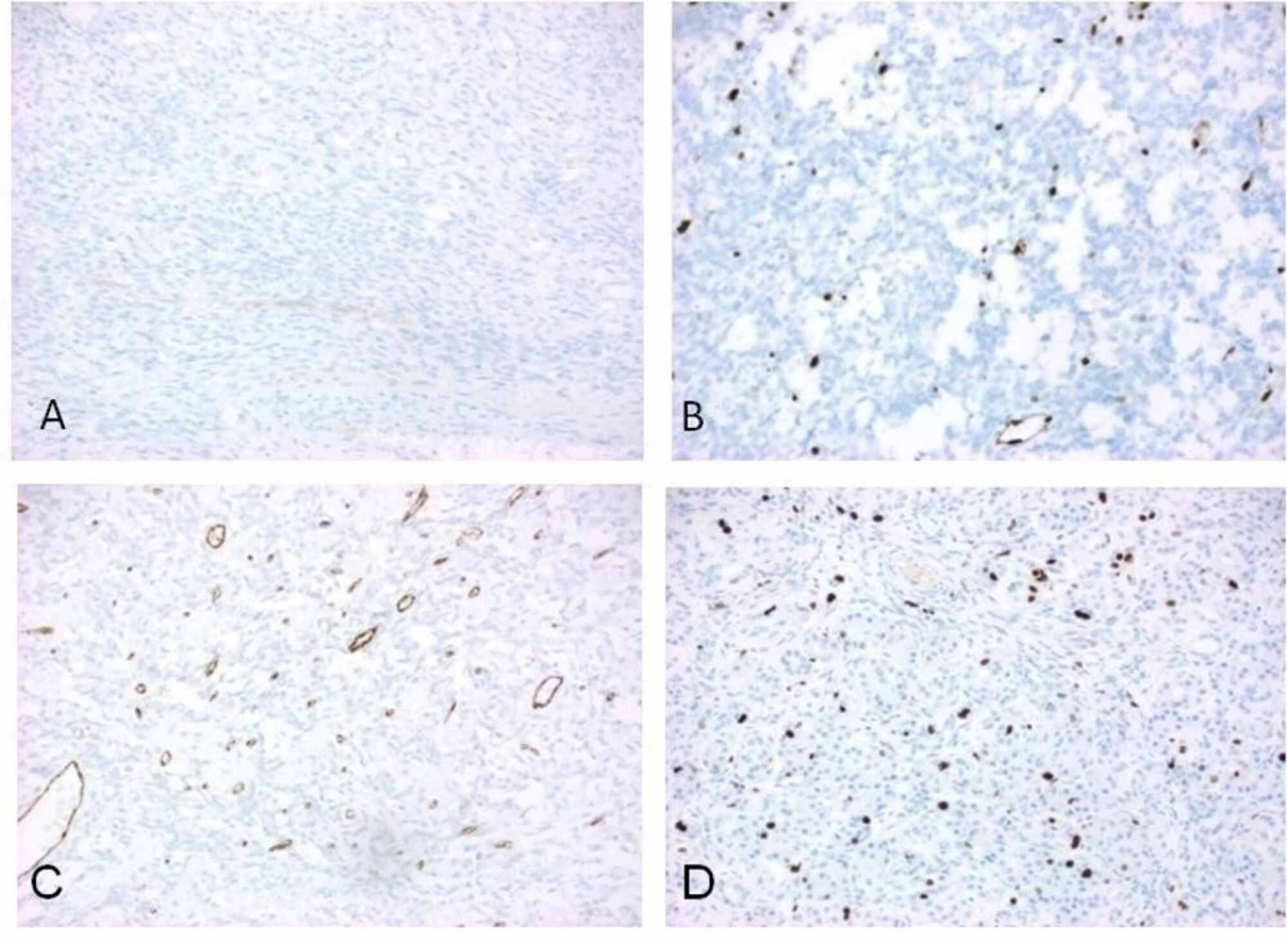
The tumor reveals DOG 1 negativity (A), thus ruled out GIST. ERG is negative in tumor cells (B), and CD31 is only focally positive (C). Ki-67 index demonstrates approximately 10% proliferative index in hypercellular area (D). DOG: Discovered on GIST. GIST: Gastrointestinal stromal tumors. ERG: Erythroblast transformation-specific-related gene. CD: Cluster of differentiation.

Since CD117 was positive, gastrointestinal stromal tumor (GIST) was ruled out by performing discovered on GIST (DOG) 1 (Figure 3A). Sarcomatous carcinoma was ruled out by negative pancytokeratin; the neuroendocrine tumor was ruled out by negative synaptophysin; vascular tumors were ruled out by negative erythroblast transformation-specific-related gene (ERG) and CD31 (Figures 3B, 3C); leiomyoma was ruled out by negative desmin and actin. Ki-67 demonstrated approximately 10% proliferative index in the hypercellular area (Figure 3D).

**Figure 4:**
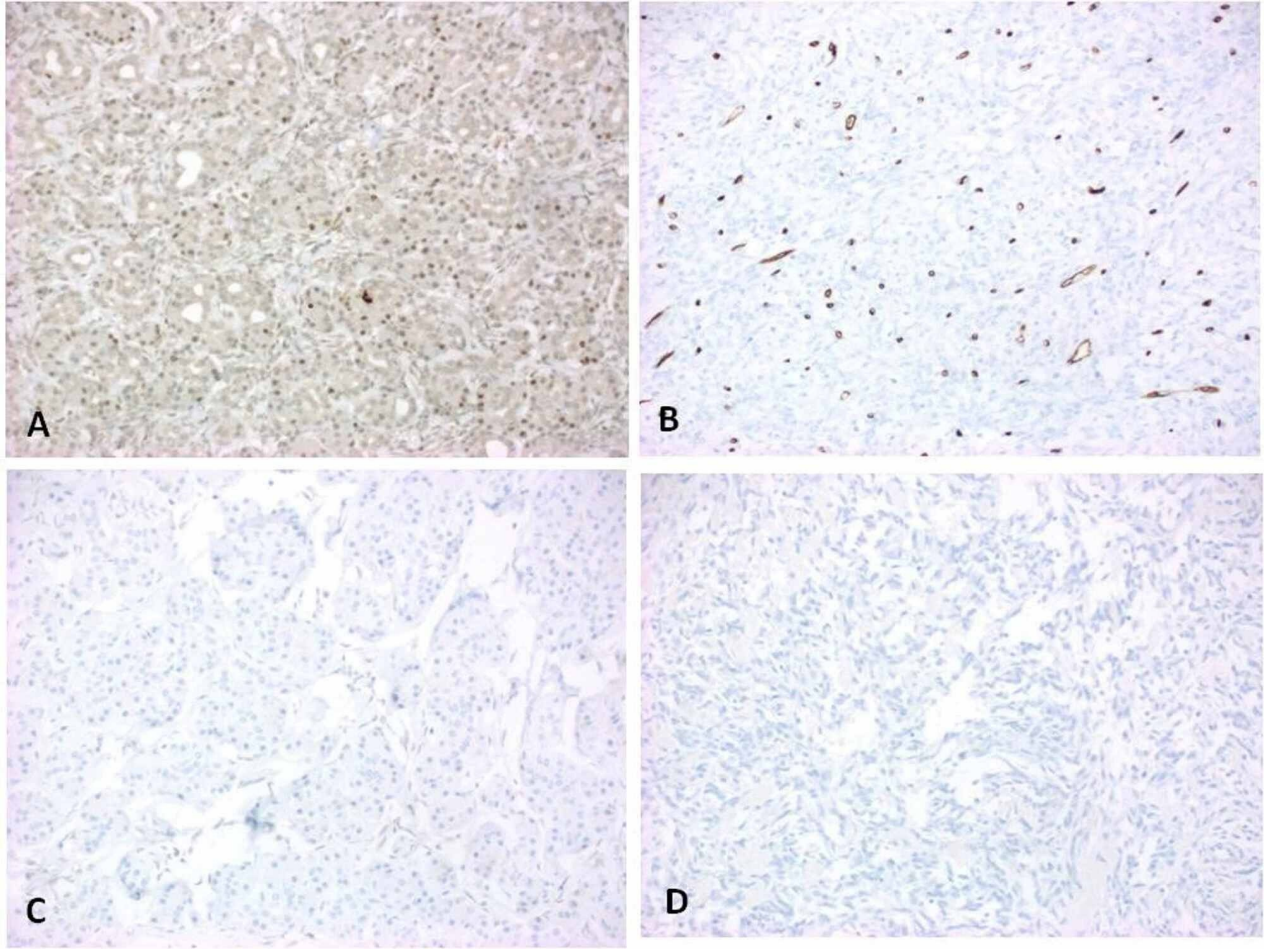
PHH3 is positive in surrounding inflammatory cells but negative in tumor cells (A). The tumor is only focally positive for CD34 (B), which is quite unusual for SFT. The tumor is negative for SOX10 (C) and S100 (D). PHH3: Phospho-histone-H3. CD: Cluster of differentiation. SFT: Solitary fibrous tumor. SOX: SRY-related HMG-box. HMG: High mobility group.

Phospho-histone-H3 (PHH3) (Figure 4A) was predominantly positive in mixed inflammatory cells with low labeling in the tumor. Desmoid fibromatosis was ruled out by negative beta-catenin and only focally positive for CD34 (Figure 4B). Melanoma was ruled out by negative SRY-related high mobility group (HMG)-box (SOX) 10 and S100 (Figures 4C, 4D). Mouse double minute 2 homolog (MDM2) by fluorescence in situ hybridization was negative in the tumor; therefore, dedifferentiated liposarcoma was ruled out.

## Discussion

The prognosis of pancreatic SFTs appears varied. Although the majority of them behave indolently, SFTs have the potential to metastasize [8-10]. Morphologic features such as cellularity, nuclear atypia, and mitotic activity, as used in the criteria for malignant SFTs of soft tissue, have prognostic values but do not always correlate with prognosis. The case presented here demonstrated hypercellularity and moderate cytologic atypia with no increased mitoses. No metastasis was discovered even when the tumor was large on the presentation.

Extrapleural SFTs are rare neoplasms that are reported in kidneys, pancreas, liver, retroperitoneum, suprarenal region, pelvis, maxillary sinus, and buccal space [1,8]. They are mostly benign with low potential of being metastatic. However, they can be aggressive with high malignant potential and can relapse after several years [9,11]. The assessment for malignant potential is necessary, and it depends on tumor size (greater than 10 cm), mitotic activity (greater than four mitotic figures per 10 high-power fields [HPFs]), the presence of necrosis or hemorrhage, cellularity, nuclear pleomorphism, and/or vascular invasion. SFTs are usually characterized by expression of STAT6, Bcl 2, CD99, and CD34 upon immunohistochemistry. They typically do not express epithelial membrane antigen (EMA) and S-100. Generally, patients with malignant diseases have an overall poor clinical course with a bad prognosis [9,10]. SFTs exhibit strong and diffuse nuclear positivity for STAT6, which differentiates them from other mesenchymal neoplasms that show both nuclear and cytoplasmic positivity [12,13]. Almost 95%-100% of SFTs are diffusely and strongly positive for CD34, but it is a non-specific marker that makes it less useful especially if the staining is patchy [1,12]. Also, CD117 positivity in SFT can be confusing as it is usually positive in GIST and negative in SFT [1,12,14]. It is, therefore, not advisable to rely on immunohistochemistry alone for making a diagnosis of GIST or SFT. As mentioned earlier, the defining criteria for malignant SFT include hypercellularity, cellular atypia, necrosis, infiltration, and greater than four mitoses per 10 HPFs [15]. Even though we found a peripherally located hypercellular area, our case does not fall into the category of malignant SFT because no necrosis or increase in mitoses was identified. It does not fall into the category of benign SFT either because of infiltrative margins, focal hypercellularity, or nuclear pleomorphism. It is, therefore, important to consider multiple factors and individualize the case if needed, in order to correctly determine management options and prognosis.

## Conclusions

The unusual or atypical morphologic and immunohistochemical features like pseudoangiomatous growth pattern, a hypercellular area demonstrating nuclear pleomorphism, and only focal positivity for CD34 can pose a diagnostic challenge especially if the morphology is almost identical to other more common pancreatic tumors like GIST. SFT should always be considered in the differential when a spindle cell lesion with no particular morphologic pattern is encountered.
